# Spatial pattern and determinants of anaemia in Ethiopia

**DOI:** 10.1371/journal.pone.0197171

**Published:** 2018-05-18

**Authors:** Bedilu Alamirie Ejigu, Eshetu Wencheko, Kiros Berhane

**Affiliations:** 1 Department of Statistics, College of Natural and Computational Sciences, Addis Ababa University, Addis Ababa, Ethiopia; 2 Division of Biostatistics, Department of Preventive Medicine, University of Southern California, Los Angeles, California, United States of America; Centers for Disease Control and Prevention, UNITED STATES

## Abstract

Anaemia is a condition in which the haemoglobin concentration falls below an established cut-off value due to a decrease in the number and size of red blood cells. The current study aimed (i) to assess the spatial pattern and (ii) to identify determinants related to anaemia using the third Ethiopian demographic health survey. To achieve these objectives, this study took into account the sampling weight and the clustered nature of the data. As a result, multilevel modeling has been used in the statistical analysis. The analysis included complete cases from 15,909 females, and 13,903 males. Among all subjects who agreed for hemeoglobin test, 5.22% males, and 16.60% females were anemic. In both binary and ordinal outcome modeling approaches, educational level, age, wealth index, BMI and HIV status were found to be significant predictors of anaemia prevalence. Furthermore, this study applied spatial methods to generate maps at regional level which could be useful for policy makers where great efforts should be concentrated to reduce the prevalence of anaemia. As revealed by Moran’s *I* test, significant spatial autocorrelation were noted across clusters. The risk of anaemia was found to vary across different regions, and higher prevalences were observed in Somali and Affar regions.

## Introduction

Anaemia is a condition in which the number and size of red blood cells, or the haemoglobin concentration falls below an established cut-off value [1, 2]. It is an indicator of both poor nutrition and health status. According to the 2011 World Health Organization (WHO) anaemia prevalence estimate, anaemia affects around 800 million children and women worldwide [2]. In low-income countries, the prevalence of anaemia remains high and is an area of priority [3]. Reducing anaemic incidence is recognized as an important component of the health of women and children, and the second global nutrition target for 2025 calls for a 50% reduction of anaemia among women of reproductive age [4].

Anaemia remains one of the biggest public health problems in Africa which is a malaria-endemic continent [5]. In Africa, the prevalence of anaemia among women of the reproductive age group 15-49 years is 37.6% [2]. Crawley [6] illustrates areas of stable malaria transmission were found more anaemic than malaria free geographical areas. Community-based estimates of anaemia prevalence among children in areas where malaria is endemic range from 49 to 76% [7].

In Ethiopia, the prevalence of anaemia is 19% and 23% for non-pregnant and pregnant women, respectively [2]. The prevalence of anaemia varies by place of residence, urban and rural; a higher proportion of women in rural areas are anaemic than those in urban areas [8]. Despite the magnitude of anaemia, geographical variability of anaemia prevalence and identification of risk factors are not well studied in Ethiopia. To our knowledge, there are no spatial studies that have reported the burden of anaemia for subjects older than 15 years old in Ethiopia.

Studies of anaemia mainly focus on pregnant women or children, among whom the burden is greatest [9–13]. In this study, we describe the spatial pattern across regions and attempt to identify risk factors of anaemia among males 15-59 years of age and females 15-49 years of age in Ethiopia. To date, studies on anaemia prevalence in Ethiopia have not assessed the spatial pattern or geographical heterogeneity [11, 14, 15]. Ignoring such geographical heterogeneity in data analysis leads to inefficient and inconsistent parameter estimates.

Spatial dependency in health-related data indicates that health outcomes in nearby neighborhoods are more similar to each other than those in distant neighborhoods. The study of geographical heterogeneity on health outcomes is modeled using multilevel or spatial mixed model [9, 10, 13]. Non-spatial multilevel modeling [16, 17] cannot address spatial dependency because the method typically assumes that neighborhoods (i.e., spatial units) are statistically independent of each other, thus multilevel models have been criticized as non-spatial and unrealistic [18–20].

The present study aims to examine spatial patterns and identify determinants of anaemia in Ethiopia using the 2011 Ethiopian Demographic and Health Survey (EDHS 2011) data. First, the study examines whether there is a significant global spatial autocorrelation for the prevalence of anaemia. If the existence of global spatial dependency is confirmed, the subsequent objective will be to explore local spatial autocorrelation, and map the spatial distribution of anaemia prevalence by region. The generated anaemia prevalence map would have important implications to targeting policy for better intervention, and to identify variables that might account for the observed spatial patterns.

Thus, the main contribution of this study would be mapping the spatial distribution of anaemia prevalence by the survey cluster and regions of the country. Further, the study would be the first ever to map anaemia for both males and females aged 15 years and older in Ethiopia. Moreover, to identify predictors of anaemia, a multilevel analysis will be done by taking into account the sampling weight and clustered nature of the dataset.

## Materials and methods

### Data

The data for this study were taken from the 2011 Ethiopian Demographic and Health Survey (EDHS 2011). The 2011 Ethiopia Demographic and Health Survey is the third comprehensive and nationally representative survey conducted in Ethiopia as part of worldwide Demographic and Health Surveys project. The main objective of the 2011 EDHS was to provide timely and reliable data on health and demographic outcomes at both national and regional levels [8]. The EDHS 2011 data were downloaded from the DHS website (http://dhsprogram.com) after being granted permission. More detailed information on DHS survey design and anaemia testing data has been summarized in [8]. The 2011 EDHS samples were selected using a stratified, two-stage cluster sampling design. In the first stage, 624 clusters of census enumeration areas (EAs), 187 in urban and 437 in rural areas, were included in the survey. Among 17,817 representative selected households in the 2011 EDHS, only complete cases from 15,909 females aged 15-49 years and 13,903 males aged 15-59 years were used. For analysis purpose, potential predictor variables such as wealth index, educational level, HIV status, BMI, age, residence (urban vs rural), region where the respondent resided, and other variables (i.e. pregnancy status) were extracted from the dataset.

Further, since malaria is one of the risk factors of anaemia ([21–24]), *Plasmodium falciparum* parasite rate (*PfPR*) to the considered DHS clusters were extracted from malaria atlas project (www.map.ox.ac.uk). Malaria Atlas Project (MAP) is one of the largest and most contemporary spatial database for *Plasmodium falciparum* parasite rate [25]. The details of the malaria data and statistical procedures for mapping it have been described elsewhere [26]. MAP presents age standardized to 2–10 years old *PfPR* which provides a valid estimate of the transmission intensity of malaria in that cluster [25]. To incorporate *PfPR* in our analysis, first we extracted geo-referenced clusters with non-zero values of *PfPR* from MAP, and assign those non-zero *PfPR* values to each individual located in nearby geo-referenced clusters.

The response variable in this study is haemoglobin level in the blood, a key indicator for anaemia. The raw measured values of haemoglobin were obtained using the HemoCue instrument and adjusted for altitude and smoking status [27]. Different cut-off points of haemoglobin level for different age groups were used to classify an individual as anemic [28]. WHO recommends specific hemoglobin levels below which an individual is specified as having anaemia, namely mild (10.0 to 11.9 g/dL), moderate (7.0 to 9.9 g/dL), and severe (<7.0 g/dL), and “any anaemia” corresponding to < 12.0 g/dL [1, 8]. Further, to see the effect of covariates on anaemia, the outcome variable for the *i*^*th*^ individual in the *j*^*th*^ *cluster* (*Y*_*ij*_) is dichotomized as follows:

$$y_{ij}=\{\begin{array}{llll} 1 & if & haemoglobin\;level<12.0g/dL & \left(Anaemic\right)\\ 0 & if & haemoglobin\;level>12.0g/dL & \left(Non-anaemic\right) \end{array}$$

Since the analysis based on the above dichotomization cannot provide information about the status of anaemia level, further discrimination which takes into account the anaemia level was done based on the following categorization [1, 28]:

$$y_{ij}=\{\begin{array}{ll} Non\;anaemic & if\;haemoglobin\;level>11.9\;g/dL\\ Mild\;anaemic & if\;haemoglobin\;level\;10.0\;to\;11.9g/dL\\ Moderate\;anaemic & if\;haemoglobin\;level\;7.0\;to\;9.9g/dL\\ Severe\;anaemic & if\;haemoglobin\;level<7.0\;g/dL \end{array}$$

The number of female and male respondents per cluster ranged from 4 to 63 with an average of 27, and 3 to 78 with an average of 23, respectively. The number of anaemic females per cluster ranges from 0 to 20 with average 5, and 0 to 10 with average 2 for males.

### Spatial autocorrelation

Spatial autocorrelation measures offer additional insight into the interdependence of spatial data. These measures quantify the correlation of a spatial random field Y(s) with itself at different locations [29]. They can be very useful to obtain information at exact locations (point-referenced data) or measurements that characterize area type data (areal data). Different statistics have been developed to test for the presence and magnitude of spatial association among areal units [30]. These include global distance-based measures such as Moran’s *I*, and Geary’s *C* (see [30–32] for discussion). The presence of spatial dependence is tested using Moran’s *I* statistic [33].

$$\begin{array}{c} I=\frac{n\sum_{i}^{n}\sum_{j}^{n}w_{ij}\left(Y_{i}-\bar{Y}\right)\left(Y_{j}-\bar{Y}\right)}{\left(\sum_{i}^{n}\sum_{j}^{n}w_{ij}\right)\sum_{i}{\left(Y_{i}-\bar{Y}\right)}^{2}} \end{array}$$ (1)

where *Y*_*i*_ represents the vector of observations at n different locations, and *w*_*ij*_ are elements of a spatial weight matrix.

Values of Moran’s *I* are assessed by a test statistic (the Moran’s *I* standard deviate) which indicates the statistical significance of spatial autocorrelation in model residuals. In this study, Moran’s *I* is calculated after we aggregate the number of anaemic subjects by survey cluster.

### Multilevel analysis

Typically, different surveys contain multiple levels of nesting. When analyzing such datasets, a multilevel model is generally more appropriate than an ordinary single-level regression model because it enables one to deal with the hierarchical structure of variables [16, 17].

A majority of demographic and health survey (DHS) sample including the one considered in this study are representative samples randomly selected from the target population. Each interviewed unit represents a certain number of similar units in the target population. Thus, to draw a valid inference from such types of surveys, the representativeness of the sample must be taken into account [34–36]. When estimating multilevel models that are based on such surveys, sampling weights are incorporated into the likelihood [36]. In this analysis, sampling weight was taken into account in both the binary and ordinal modeling approaches.

The multilevel model assumes that individual-level (i.e., lower hierarchy) observations belonging to a particular cluster (i.e., higher hierarchy) are not independent of each other because they share similar characteristics of that cluster [16, 17]. Multilevel analysis is needed as analytic means because the nested structure of the data requires simultaneous examination of cluster and individual-level variables [37]. The multilevel approach produces reliable standard errors and parameter estimates when outcomes for individuals within clusters are correlated [17]. Multilevel models consist of two sets of equations: one explaining variation at individual level, and the other explaining variation at cluster level.

In this study two different multilevel models were fitted: (1) multilevel logistic regression with a dichotomous dependent variable (anaemic versus not anaemic); and (2) multilevel ordinal logistic regression (severe, moderate, mild, not anaemic).

1. **Multilevel Generalized Linear Models for Binary Outcome**
  The multilevel logistic regression model is a very popular choice for analysis of dichotomous data. Due to the fact that the probability of having anaemia possibly varies in different clusters, a cluster-level random intercept is introduced in the generalized linear mixed model. Let *y*_*ij*_ denote the binary outcome for subject *i* in cluster *j*, and assume *y*_*ij*_ follows a Bernoulli distribution with probability of success (in our case anaemic), *p*_*ij*_. Then, using the usual logit link function, a binary outcome can be associated with a linear predictor as follows:
  $$\begin{array}{c} logit\left(p_{ij}\right)=\beta_{0}+\beta X_{ij}+u_{j} \end{array}$$ (2)
  where *β*_0_ is an intercept, *β* is an unknown parameter for individual level predictors, and *u*_*j*_ are mutually independent Gaussian random effects used to capture within-cluster correlation. In standard multilevel models, *u*_*j*_ is usually assumed to be a normally distributed random intercept with mean 0 and variance $\sigma_{u}^{2}$ [17].
  To test whether the variance of the random intercept is significant (*H*_0_: *d* = *d*_0_ = 0 against *H*_*a*_: *d* > 0), the likelihood ratio test was applied in which under *H*_0_ the sum $-2ll\left(d_{0}\right)+2ll\left(d\right)∼\frac{1}{2}\chi^{2}\left(0\right)+\frac{1}{2}\chi^{2}\left(1\right)$. In the expression 2*ll*(*d*_0_) stands for the value of the loglikelihood function related to the model under *H*_0_ whereas 2*ll*(*d*) equals the value of the log-likelihood function for the GLMM under consideration.
  When the logistic model is applied, the level-one residuals are assumed to follow the standard logistic distribution, with mean 0 and variance *π*^2^/3 ≈ 3.29. This variance represents the within-group variance for intraclass correlation (ICC) for dichotomous data; ICC can be similarly defined for ordinal outcomes [38]. For Model 1, the intraclass correlation is: $ICC=\frac{\sigma_{u}^{2}}{\sigma_{u}^{2}+3.29}$.
2. **Multilevel Generalized Linear Models for Ordinal Outcome**
  Using ordered outcomes yields more parsimoniously parameterized models. A common tool for analyzing regression data with ordinal responses is the cumulative threshold model [39]. The model assumes that the response variable *Y*_*ij*_, here anaemia status for subject *i* in cluster *j*, is a categorized version of a latent continuous variable, say individual haemoglobin level (as in this study). The model is
  $$\begin{array}{c} logit\left(P\left(Y_{ij}<k|X_{ij}\right)\right)=\beta_{0k}+\beta X_{ij}+u_{j} \end{array}$$ (3)
  where $u_{j}∼N\left(0,\sigma_{u}^{2}\right)$ as in Eq (2).

The weighted multilevel analysis was done using Stata [40]. Spatial maps of anaemia prevalence by cluster and region, and spatial autocorrelation tests were done using ArcGIS 10.5 [41].

## Results

In this section, the data introduced earlier are analyzed, and results of the analysis based on multilevel and spatial data analysis techniques will be presented. Recall that the aims of the study are to assess the spatial pattern and identify determinants of anaemia in Ethiopia for females and males aged 15 years and older.

### Descriptive results

Table 1 shows prevalence of anaemia for males and females by region. The results reveal that in all regions anaemia prevalence is higher among females than males. Higher prevalence of anaemia was observed in Somali and Affar regions for both genders. The lowest prevalence was observed in Addis Ababa and SNNP region (Table 1).

**Table 1:** Percentage of anaemia at different levels by region among males and females.

Region	Female			Male		
	Severe	Moderate	Mild	Severe	Moderate	Mild
Tigray	0.38(0.18,0.81)	2.33(1.70,3.18)	9.81(8.48,11.33)	0.09(0.01,0.63)	1.44(0.94,2.19)	3.98(3.11,5.09)
Affar	1.43(0.88,2.29)	9.46(7.86,11.35)	23.59(21.02,26.37)	0.18(0.05,0.61)	1.13(0.60,2.13)	4.46(3.28,6.05)
Amhara	0.36(0.18,0.74)	2.33(1.76,3.08)	13.77(12.31,15.37)	0.04(0.01,0.31)	0.85(0.53,1.35)	4.87(4.02,5.89)
Oromiya	0.61(0.35,1.06)	3.29(2.60,4.16)	15.27(13.76,16.91)	0.19(0.07,0.52)	1.20(0.81,1.78)	4.62(3.78,5.63)
Somali	4.50(3.15,6.37)	15.01(12.45,17.97)	24.66(21.47,28.17)	0.11(0.02,0.79)	2.08(1.16,3.70)	4.79(3.32,6.86)
Ben-Gumiz	0.46(0.17,1.23)	4.19(3.11,5.63)	14.36(12.44,16.52)	0.63(0.26,1.55)	2.39(1.58,3.61)	4.59(3.47,6.06)
SNNP	0.36(0.17,0.77)	2.17(1.61,2.92)	8.72(7.54,10.07)	0.21(0.08,0.57)	0.64(0.35,1.15)	2.24(1.62,3.08)
Gambela	0.23(0.07,0.67)	3.44(2.33,5.03)	15.40(12.77,18.45)	.	0.78(0.26,2.36)	3.73(2.38,5.79)
Harari	0.74(0.35,1.55)	4.33(3.23,5.77)	14.23(12.19,16.56)	1.15(0.62,2.15)	1.68(1.07,2.79)	2.67(1.79,3.97)
Addis Ababa	0.28(0.11,0.67)	1.17(0.75,1.83)	7.92(6.69,9.35)	.	0.21(0.05,0.83)	0.99(0.57,1.72)
Dire Dawa	1.43(0.88,2.30)	9.92(8.27,11.85)	17.27(15.05,19.75)	0.77(0.38,1.54)	1.67(1.01,2.73)	4.90(3.65,6.55)

Table 2 provides a summary of the percentage of anaemia by gender under different categorical covariates including educational level, age, residence, HIV status, BMI, *PfPR* and wealth index. For both genders higher prevalence of anaemia was observed in rural areas compared with individuals who live in urban areas. Furthermore, anaemia prevalence decreases as wealth index and educational level increase for both genders. For females, the prevalence of anaemia is higher when age is above 18, while for males the prevalence is higher among younger males. To see whether this difference is statistically significant or not, a multilevel model which includes all potential predictors simultaneously has been fitted. The results are presented in Tables 3 and 4.

**Table 2:** Prevalence of anaemia with different levels of anaemia by background characteristics for females and males.

Factors	Female			Male		
	Severe	Moderate	Mild	Severe	Moderate	Mild
**Residence**						
Urban	0.23(0.12,0.44)	1.94(1.42,2.64)	8.92(7.68,10.33)	0.13(0.27,0.61)	0.28(0.13,0.61)	1.69(1.13,2.53)
Rural	0.64(0.46,0.87)	3.25(2.83,3.72)	14.35(13.46,15.29)	0.15(0.08,0.29)	1.18(0.93,1.51)	4.65(4.12,5.25)
**Education label**						
No education	0.80(0.58,1.12)	3.4(2.94,4.04)	15.93(14.81,17.13)	0.27(0.12,0.59)	1.36(0.95,1.94)	5.35(4.49,6.35)
Primary	0,27(0.14,0.55)	2.88(2.33,3.56)	10.68(9.58,11.88)	0.03(0.01,0.12)	0.98(0.071,1.35)	4.18(3.55,4.91)
Secondary	0.28(0.06,1.20)	0.64(0.33,1.23)	8.17(6.08,10.89)	0.47(0.14,1.57)	0.50(0.18,1.37)	1.12(0.55,2.30)
Higher	0.20(0.05,0.83)	1.11(0.54,2.24)	8.47(5.69,12.44)	-	0.11(0.02,0.48)	0.63(0.17,2.31)
**Age**						
<18	0.19(0.05,0.65)	1.78(1.18,2.68)	10.72(8.94,12.81)	0.26(0.06,1.07)	1.12(0.67,2.11)	6.72(5.23,8.59)
≥18	0.60(0.45,0.81)	3.15(2.76,3.59)	13.53(12.71,14.39)	0.13(0.07,0.13)	0.98(0.76,1.26)	3.68(3.23,4.23)
**Wealth index**						
Poorerest	0.98(0.59,1.62)	4.04(3.20,5.11)	14.97(13.29,16.81)	0.34(0.14,0.78)	1.44(0.91,2.27)	6.30(5.10,7.83)
Poorer	0.46(0.22,0.97)	2.98(2.24,3.96)	15.29(13.49,17,29)	0.23(0.06,0.86)	1.78(1.17,2.70)	4.56(3.55,5.83)
Middle	0.82(0.45,1.48)	2.69(1.97,3.66)	13.48(11.73,15.46)	0.09(0.02,0.34)	0.87(0.50,1.49)	4.47(3.48,5.72)
Richer	0.38(0.18,0.79)	3.60(2.7.29,4.73)	13.47(11.72,15.45)	0.01(0.00,0.02)	0.88(0.51,1.48)	3.71(2.82,4.87)
Richest	0.19(0.09,0.37)	1.76(1.30,2.40)	9.47(8.22,10.89)	0.12(0.03,0.53)	0.26(0.12,0.55)	1.90(1.33,2.72)
BMI						
<18.5	0.72(0.43,1.23)	3.29(2.59,4.16)	14.60(13.10,16.25)	0.12(0.04,0.33)	1.46(1.08,1.99)	5.74(4.90,6.71)
18.5-24.9	0.52(0.36,7.37)	2.90(2.49,3.38)	12.92(12.01,13.89)	0.15(0.07,0.34)	0.72(0.50,1.03)	3.19(2.68,3.78)
>24.9	0.04(0.01,0.14)	2.05(1.25,3.33)	9.13(6.98,11.85)	0.43(0.08,2.23)	1.28(0.33,4.92)	1.26(0.32,4.78)
HIV						
Positive	0.48(0.15,1.51)	3.44(1.31,8.70)	16.29(11.21,23.06)	0.04(0.01,0.26)	1.34(0.46,3.80)	6.56(2.73,14.96)
Negative	0.54(0.41,0.73)	2.94(2.59,3.33)	13.03(12.27,13.83)	0.14(0.08,0.27)	1.00(0.79,1.26)	4.05(3.60,4.55)
Malaria						
*PfPR* < 0.005	0.43(0.30,0.64)	2.63(2.21,3.13)	13.33(12.32,14.41)	0.15(0.06,0.33)	0.97(0.71,1.31)	3.87(3.30,4.52)
0.005 ≤ *PfPR* < 0.015	0.34(0.04,2.37)	3.13(1.79,5.44)	10.62(7.95,14.04)	-	0.44(0.11,1.77)	2.54(1.32,4.84)
0.015 ≤ *PfPR* < 0.025	0.38(0.05,2.68)	0.81(0.20,3.23)	20.06(11.54,32.55)	-	0.51(0.07,3.61)	5.87(2.08,15.51)
*PfPR* ≥ 0.025	-	0.75(0.27,2.09)	5.52(3.34, 8.99)	-	0.03(0.007,0.12)	1.30(0.53,3.44)

- no observation

**Table 3:** Adjusted odds ratios (AOR) and 95% CI of adjusted odds ratios (AOR) for multilevel mixed-effects binary logistic regression model.

Factors	Female		Male	
	AOR	95% CI for AOR	AOR	95% CI for AOR
$\hat{\beta_{0}}$*	0.15	(0.09, 0.23)	0.11	(0.06, 0.19)
**Residence** (ref: Urban)				
Rural	1.4	(0.99, 1.96)	1.49	(0.89,2.48)
**Education** (ref: no education)				
Primary	0.74	(0.64,0.86)	0.74	(0.58,0.94)
Secondary	0.55	(0.40, 0.75)	0.41	(0.21, 0.82)
Higher	0.64	(0.43, 0.92)	0.12	(0.04, 0.35)
**Age** (ref: <18)				
≥ 18	1.33	(1.07,1.65)	0.65	(0.48,0.89)
**Pregnancy** (ref: not pregnant)				
Pregnant	1.43	(1.15,1.78)	-	-
**Wealth index** (ref: poorest)				
Poorer	0.96	(0.78,1.18)	0.79	(0.56, 1.12)
Middle	0.89	(0.72,1.11)	0.71	(0.52,0.98)
Richer	0.93	(0.75, 1.15)	0.64	(0.46,0.90)
Richest	0.89	(0.64,1.25)	0.61	(0.36, 1.04)
BMI (ref: <18.5)				
18.5–24.9	0.83	(0.72, 0.96)	0.60	(0.48, 0.74)
≥24.9	0.60	(0.44, 0.83)	0.59	(0.26,1.34)
HIV (ref: negative)				
Positive	2.09	(1.41, 3.08)	3.56	(1.51,8.36)
Malaria (ref: *PfPR* < 0.005)				
0.005 ≤ *PfPR* < 0.015	0.84	(0.62, 1.15)	0.55	(0.33, 0.93)
0.015 ≤ *PfPR* < 0.025	1.79	(0.82, 3.88)	2.02	(1.29, 3.15)
*PfPR* ≥ 0.025	0.71	(0.26, 1.86)	0.44	(0.19, 0.98)
**Random intercept**				
Var(*u*_*j*_)	0.36	(0.27, 0.48)	0.40	(0.25, 0.63)

* result presented in log odds, not in odds ratio.

**Table 4:** Adjusted odds ratios(AOR) and 95% CI of adjusted odds ratios for the multilevel mixed-effects ordinal logistic regression model.

Factors	Female		Male	
	AOR	95% CI for AOR	AOR	95% CI for AOR
**Intercepts**				
*cut*1*	1.92	[1.49, 2.35]	2.24	[1.64, 2.83]
*cut*2*	3.69	[3.24, 4.14]	3.83	[3.22, 4.46]
*cut*3*	5.6	[5.08, 6.13]	5.86	[5.01, 6.71]
**Residence** (ref: urban)				
Rural	1.14	[1.001,1.96]	1.48	[0.89, 2.46]
**Education label** (ref: no education)				
Primary	0.76	[0.65,0.88]	0.74	[0.58, 0.93]
Secondary	0.55	[0.41,0.75]	0.41	[0.20,0.83]
Higher	0.64	[0.40,0.93]	0.12	[0.04, 0.35]
**Age** (ref: <18)				
≥ 18	1.34	[1.08,1.66]	0.66	[0.48, 0.89]
**Pregnancy** (ref: not pregnant)				
Pregnant	1.61	[1.27,2.03]		
**Wealth index** (ref: poorest)				
Poorer	0.95	[0.78, 1.67]	0.79	[0.56, 1.12]
Middle	0.91	[0.73, 1.23]	0.70	[0.51, 0.96]
Richer	0.93	[0.75, 1.15]	0.64	[0.45, 0.89]
Richest	0.88	[0.64, 1.23]	0.60	[0.35, 1.02]
**BMI** (ref: <18.5)				
18.5–24.9	0.82	[0.72, 0.95]	0.60	[0.47, 0.74]
>24.9	0.59	[0.43, 0.84]	0.62	[0.26, 1.47]
**HIV** (ref: negative)				
Positive	2.09	[1.41, 3.11]	3.56	[1.55, 8.18]
Malaria (ref: *PfPR* < 0.005)				
0.005 ≤ *PfPR* < 0.015	0.85	(0.62, 1.16)	0.55	(0.33, 0.92)
0.015 ≤ *PfPR* < 0.025	1.70	(0.81, 3.58)	1.94	(1.23, 3.08)
*PfPR* ≥ 0.025	0.68	(0.26, 1.83)	0.43	(0.19, 0.97)
**Random intercept**				
Var(*u*_*j*_)	0.42	[0.31,0.57]	0.42	[0.26,0.65]

* result presented in log odds, not in odds ratio.

**Fig 1:**
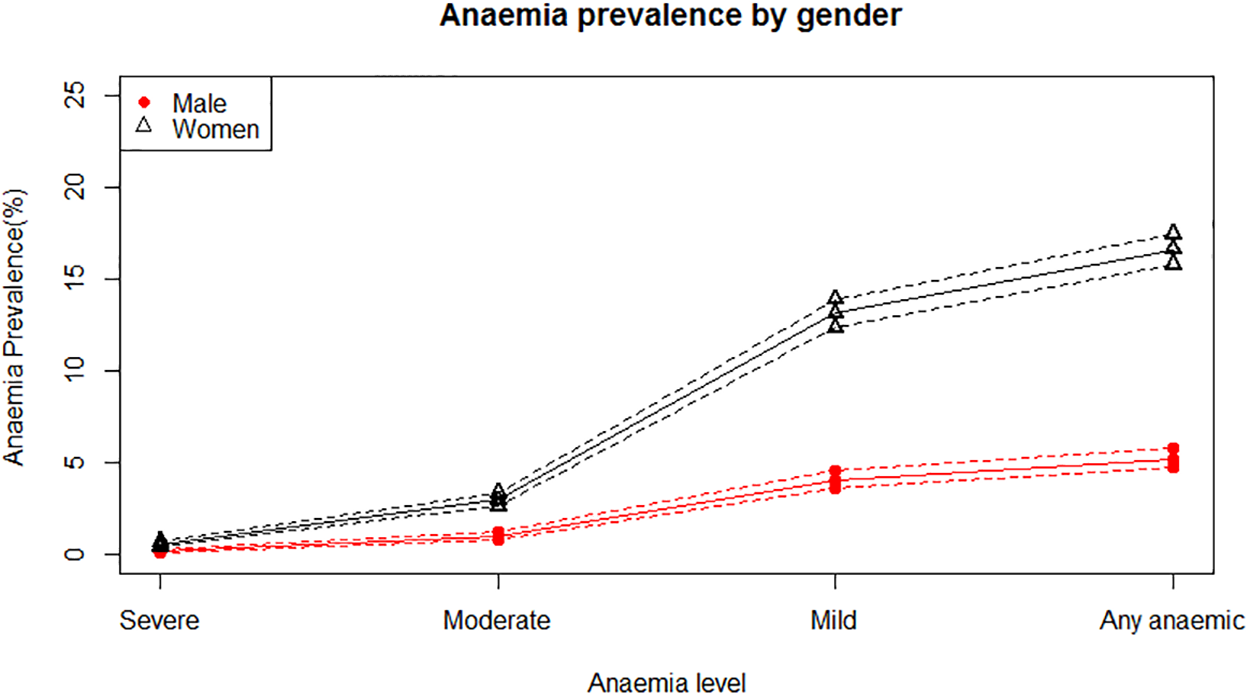
Different levels of anaemia prevalence by gender for the 2011 EDHS data.

Fig 1 presents the prevalence of anaemia at the various levels of anaemia by gender. The dotted line represents 95% confidence interval for the estimated prevalence of anaemia at different levels. The result reveals that, females are more anaemic than males.

### Spatial data analysis

**Fig 2:**
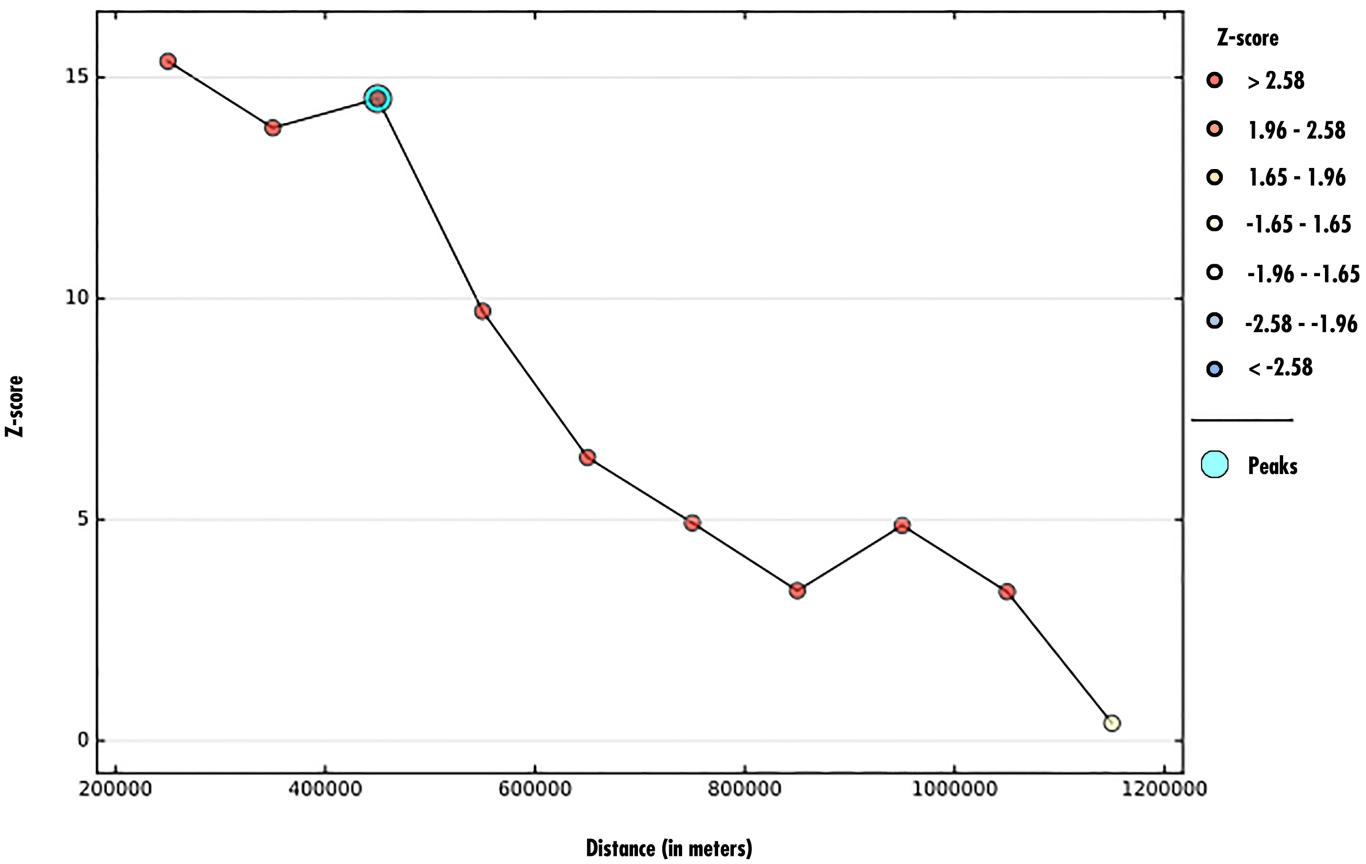
Spatial autocorrelation as a function of distance.

To get a general insight into the spatial clustering of anaemia, a global spatial statistic was estimated using Moran’s *I* statistic (Eq 1). This was done after establishing the number of anaemia cases in each of the clusters. The test result showed the presence of significant global positive spatial autocorrelation for the prevalence of anaemia (for males *I* = 0.12, P-value < 0.0001, and females *I* = 0.15, P-value < 0.0001). The global Moran’s *I* statistic result suggests that there is local clustering in the distribution of anaemia prevalence that need to be further explained using local spatial statistics. Fig 2 presents graphical plots of the Moran’s *I* test statistic values as a function of distance (in meters). As presented in Fig 2, significant local clustering of anaemia prevalence occurs between pairs of clusters within 1-400 km distance lags; the highest mean local Moran’s *I* values are observed at 200 km distance lag.

**Fig 3:**
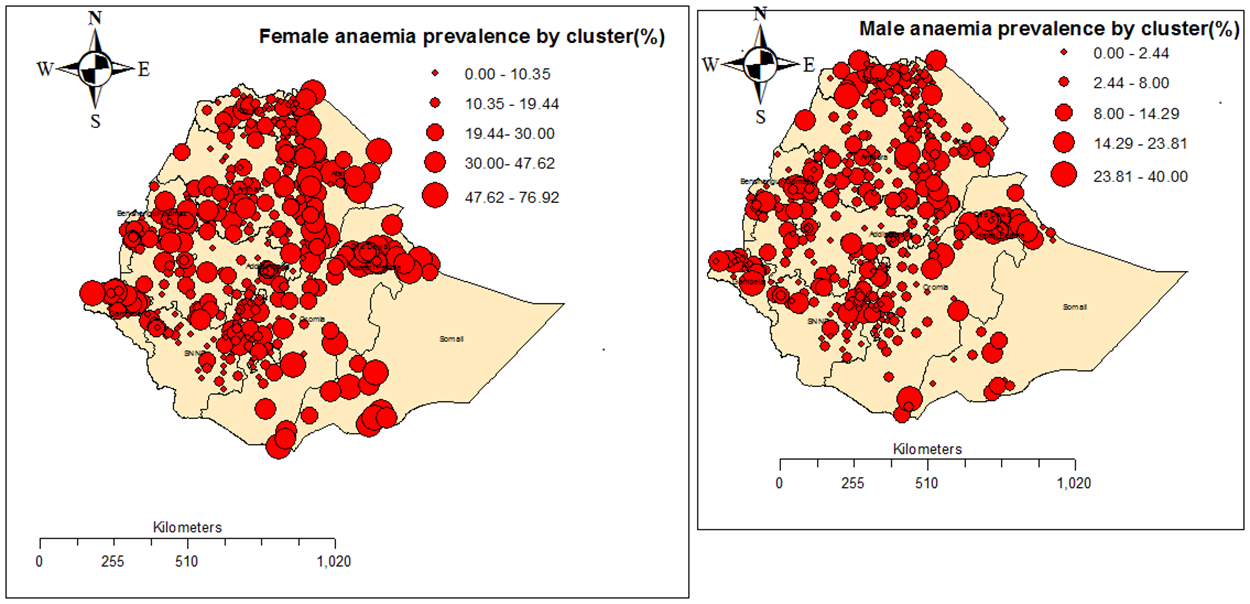
Observed anaemia prevalence for the 2011 EDHS survey clusters.

Fig 3 below presents the observed anaemia prevalence by the 2011 EDHS survey clusters. The result shows that the prevalence of anaemia varies from cluster to cluster.

**Fig 4:**
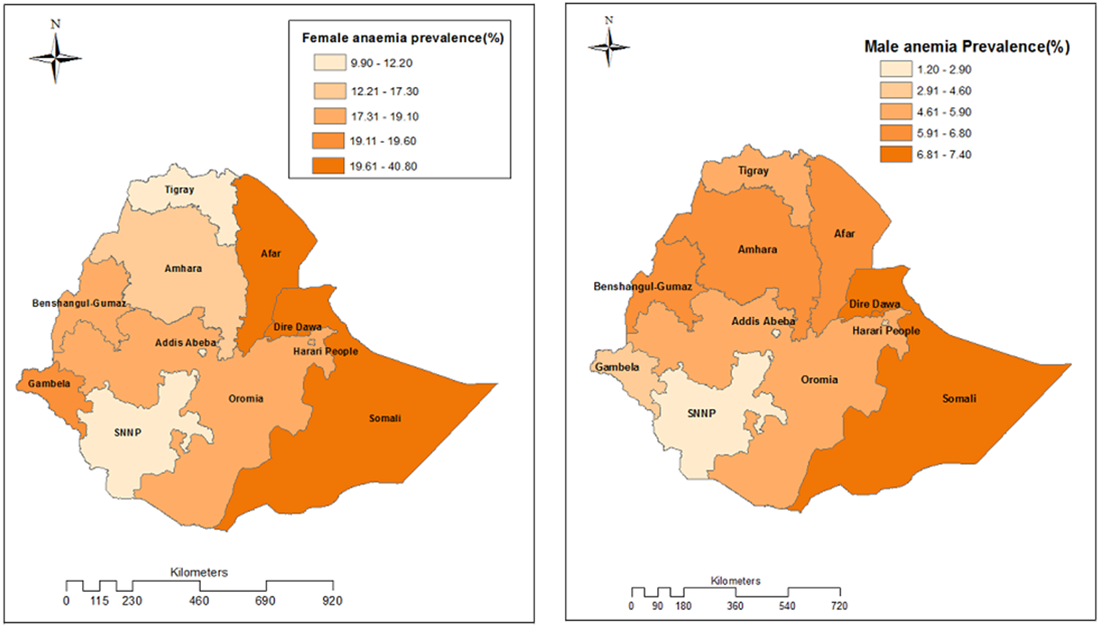
National estimates of the prevalence of anaemia in Ethiopia for the 2011 EDHS data.

To get a general insight on the prevalence of anaemia by regions of the country, Fig 4 presents the prevalence of anaemia by regions of the country. The map reveals that the eastern part of the country had higher anaemia prevalence than the south-west region. As presented in Table 1 above, the prevalence was highest in Somali (44.16% (95% CI:40.37,48.01) for females, 7.5% for males) and then for the Affar region (34.48% (95% CI:31.59.37,48.01) for females, 6.8% for males), and lowest in Addis Ababa (9.37% (95% CI:8.04,10.90) for females, 1.3% for males) and SNNP region (11.26% (95% CI:9.92,12.75) for females, 3.2% for males).

**Fig 5:**
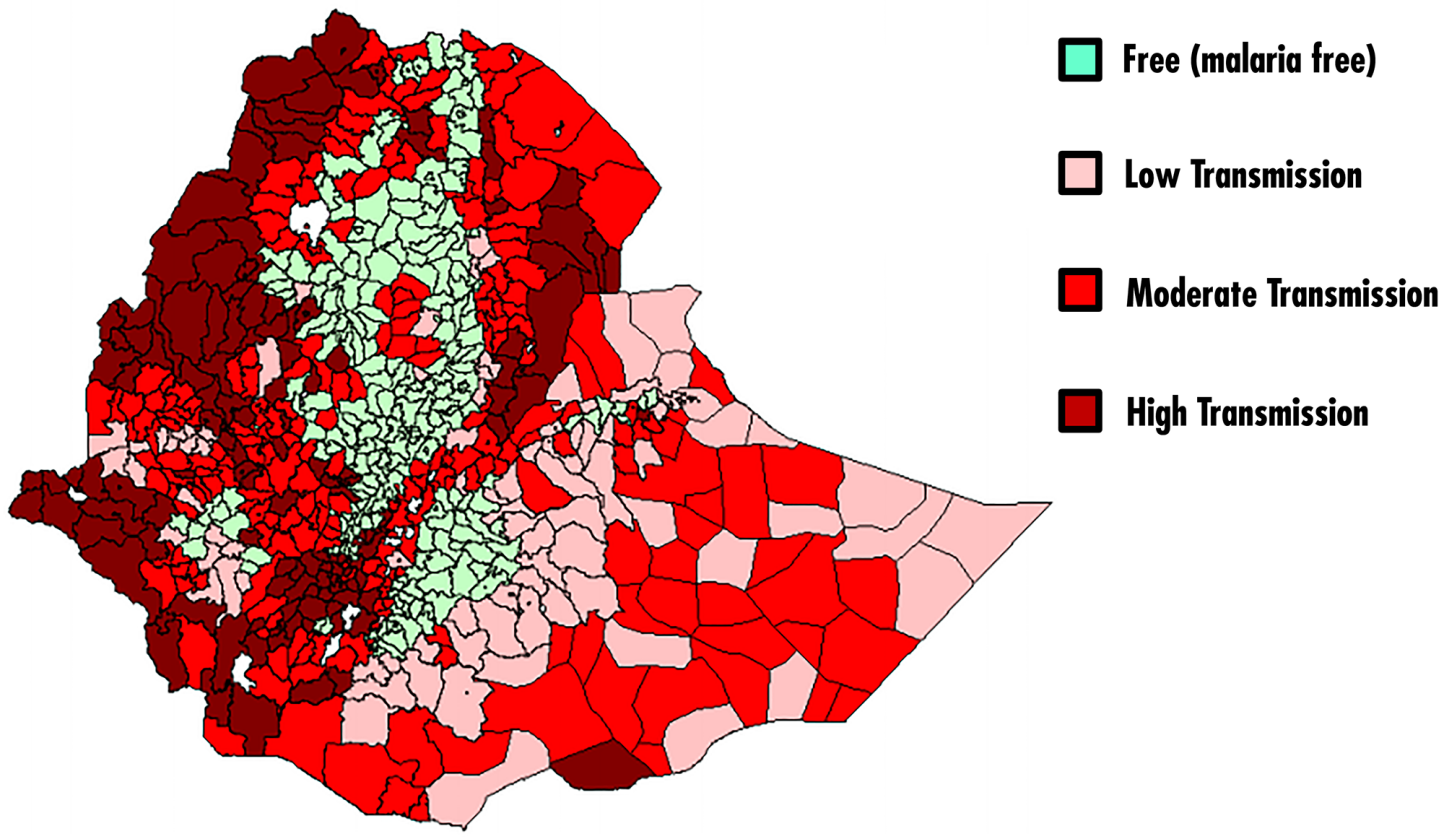
Risk map of districts by annual malaria parasite incidence, Ethiopia (Source: FMOH [43]).

Fig 5 presents the risk map of districts by annual malaria parasite incidence in Ethiopia. The Figure reveals that the central part of the country is malaria free. Nowadays, the overall malaria prevalence in Ethiopia is very low [42]. Malaria parasite prevalence in areas <2,000m was 0.5 percent by microscopy blood-slide examination for all ages and 0.6 percent among children under 5 year [42]. Among many other factors, malaria plays a major causative role of anaemia globally. The malaria-attributable fraction of anaemia may then differ in different settings. In this study, the spatial pattern of anemia and malaria (Figs 3 and 4) is not similar.

### Multilevel mixed-effects binary logistic regression model

As mentioned in the previous section, by considering sampling weights multilevel logistic regression models can be employed to identify potential predictors related to anaemia. The interpretations of the results here are given along adjusted odds-ratio as shown in Table 3. The results reveal that educational level, wealth index, BMI, HIV status, and age had significant effect on the prevalence of anaemia. As demonstrated in [14, 44–46], being pregnant significantly raises the likelihood of being anaemic. The current study shows that pregnant women were 1.43 times (43% more) likely to be anaemic than non-pregnant women. The effect of age on the prevalence of anaemia is different for males and females. The chance of being anaemic for females above 18 years of age was 1.33 times higher (33% more likely) than those below 18 years of age. Males older than 18 years of age were 35% less likely to be anaemic than those younger than 18 years. The place of residence (urban or rural) where the subject lives does not have any impact on the prevalence of anaemia. HIV positive females are 2.09 times more likely to be anaemic than HIV negative females. HIV positive males were 3.56 times more likely to be anaemic than HIV negative males.

Females with BMI between 18.5 and 24.9 were 17% less likely to be anaemic than females with lower BMI (BMI <18.5). Further, the likelihood of being anaemic for overweight (BMI >24.9) females was 40% less likely than underweight females. There was no statistically significant difference between underweight and overweight males in the likelihood of being anaemic. But, males with BMI 18.5-24.9 were 40% less likely to be anaemic than underweight (BMI < 18.5) males.

In the binary outcome model, for males who live in areas with *Plasmodium falciparum* parasite rate between 0.015 and 0.025 (0.015 ≤ *PfPR* < 0.025) the estimated odds of being anaemic had increased than for males who live in areas with low *Plasmodium falciparum* parasite rate (*PfPR* < 0.005) [estimated odds ratio (OR) 2.02, 95% confidence interval (CI) 1.29—3.15]. Due to few number of *PfPR* data extracted from MAP, statistically significant association between anaemia and areas with apparent Plasmodium falciparum parasite were not observed for females (Table 3).

Under the binary multilevel logistic model (Eq 1), the intraclass correlation was equal to $\frac{0.40}{0.40+3.29}=0.11$, implying that two subjects located in the same cluster had a correlation equal to 0.11 to be anaemic for males.

### Multilevel mixed-effects ordinal logistic regression model

In order to quantify the effect of each one of the determinants (by taking into account the ordinal nature of the outcome variable), we considered Eq (3) together with the cluster-level random effects. The assumption of proportional odds was tested using the user-contributed command for multilevel and latent variable modeling, called gllamm package in Stata [40, 47]. The results reveal that the assumption of proportional odds is tenable at 5% level of significant for all considered covariates in the model.

Similar to the aforementioned binary logistic regression model results, educational label, wealth index, HIV, BMI, and pregnancy status were found significant determinant factors of anaemia prevalence. Parameter estimates and their standard errors, together with the corresponding adjusted odds-ratios are given in Table 4.

Pregnant women were 1.61 times (61%) more likely to be anaemic than non-pregnant women. Similar to the binary outcome model result, the effect of age on the prevalence of anaemia is different for males and females, and place of residence (urban or rural) where the subject lives does not have any impact on the prevalence of anaemia for males, and a borderline significant result was observed for females. Similar to the binary outcome model result, HIV positive females are 2.09 times more likely to be anaemic than HIV negative females, and HIV positive males were 3.56 times more likely to be anaemic than HIV negative males.

The likelihood of being anaemic for males with BMI between 18.5 and 24.9 was found to be 40% less likely than males with lower BMI (BMI <18.5). Further, the likelihood of being anaemic for overweight (BMI >24.9) males was 38% lower than underweight males. After adjusting for important covariates and by considering the ordinal nature of the outcome variable, we found that the *PfPR* of the area of residence is not statistically correlated with anaemia prevalence (Table 4) for females. This may be due to the fact that malaria prevalence is very low (less than 0.5%) in Ethiopia [42].

The estimated variances of the random effects are significant at the 0.05% level, indicating that there are substantial differences between clusters.

## Discussion

The prevalence of anaemia was found to vary geographically—higher in the eastern part of the country and lower in the south-west part (Fig 4). The observed spatial variation of anaemia across regions could be due to the regional differences in dietary preferences; infectious disease risk; access to health care centers or any other factors. The prevalence of anaemia was found to be low in SNNP region which is dominated by maize-mixed agriculture. Messina and his colleagues [13] conclude that living in a community dominated by maize-mixed agriculture was significantly associated with lower chance of being anaemic (74% less likely) as well as greater hemoglobin levels.

Although the descriptive statistics (Table 2) and the report by Central Statistical Agency of Ethiopia [8] suggest that prevalence of anaemia is associated with place of residence (urban and rural), lack of statistical significance in our model (Tables 3 and 4) indicates that other factors are more likely to account for anaemia prevalence. Urban residence was associated with a low burden of anaemia among women [13, 48].

In this study, at individual level, wealth, BMI, HIV status, educational level, pregnancy and age were identified as significant determinants of anaemia in both multilevel mixed-effects binary and ordinal logistic regression model at 5% level of significance (Tables 3 and 4). Due to the low prevalence of HIV(1.3%) in their study Messina [13] did not show any association between HIV and anaemia. While, in this study, even if the prevalence of HIV was 1.4% for males and 2.3% for females, significant association between anaemia and HIV was demonstrated in both binary and ordinal logistic regression models (Tables 3 and 4). In this study, the likelihood of being anaemic for HIV positive females was two-times higher than those HIV negative females, and it was 3.56 times higher for HIV positive males as compared with HIV negative males (Table 3). When we take into account the ordinal nature of the outcome variable, a similar result was observed with the binary outcome model(Table 3) were observed in the likelihhod of being anaemic for both HIV positive males and females (Table 4). The estimated variance of the random intercepts using the weighted analysis for cluster level are significantly different from zero, indicates considerable heterogeneity in anaemia prevalence with respect to sampling cluster that is unaccounted for by the predictor variables (Tables 3 and 4).

Unlike the findings by different scholars [5, 21–24], our results suggest that malaria is not likely to be a risk factor for anaemic individuals living in those areas with high malaria prevalence. This may be due to the fact that our work makes use of *Plasmodium falciparum* endemicity from limited number of geographical estimates for PfPR in locations where demographic and health surveys were made, and malaria prevalence is very low (less than 0.5%) in Ethiopia [42].

Carneiro [5] analyze how the prevalence of anemia depends on that of *Plasmodium falciparum* malaria by developing models of the excess risk of anemia caused by malaria at a population level in 24 villages in northeastern Tanzania. Their study result reveals that the prevalence of a hemoglobin level < 8*g*/*dL* attributable to malaria was 4.6% in infants, 4.1% in children one year of age, 2.7% in children two years of age, and 3.3% in women of childbearing age [5].

There was no statistically significant difference between underweight and overweight males in the likelihood of being anaemic. But, males with BMI 18.5-24.9 were 40% less likely to be anemic than underweight (BMI < 18.5) males. Further, when we consider sampling weights in the analysis, wealth index was not found statistically significant on the likelihood of being anaemic for females. While the likelihood of being anaemic for males with middle and richer wealth index was lower as compared with poorest, poorer and richest wealth index individuals.

The results of analysis in this study showed that the findings of the weighted analysis generally agree with the un-weighted analysis. The main difference that was observed between the weighted and un-weighted analysis was in the confidence intervals of parameter estimates. The confidence intervals were narrow for the un-weighted analysis but due to larger standard errors the confidence intervals become wider when we take into account the sampling weight in the analysis. Further, the estimated regression coefficient and standard errors from the weighted analysis diverged slightly from the un-weighted analysis (i.e. parameter estimates from the un-weighted analysis are slightly lower).

In contrast with the findings of our study and others [10, 49], the findings from Messiana [13] do not show any significance association of anaemia with body mass index and wealth. Similar to our study results, [10, 49] show a significant association between wealth and anaemia.

Supporting our findings, [50–52] found increased risk for anaemia among less educated individuals. Contrary to our study results, [13, 53, 54] found no association between anaemia and educational level. The study by Messina [13] did not account for potential between-cluster correlation in their analysis. They applied the standard multilevel models to identify predictors of anaemia in Congolese women. Studies on the spatial variation of anaemia risk for adult individuals have not been undertaken well yet [55]. The study by Soares [55] showed that malnutrition and parasitological risk factors highly contributed to the spatial variation in individual-level anaemia.

## Conclusion

In summary, our study shows that the prevalence of anaemia is associated with wealth, educational level, BMI, HIV, age and pregnancy status. Spatial variability of anaemia prevalence across survey clusters and regions were observed; it was higher in the eastern part of the country.

The main limitation of this study was the inability to incorporate other potential correlates of anaemia such as hookworm infection [56] and dietary information which is associated with anaemia [54, 57] were not included in this study.
